# Keep up or drown: adjustment of western Pacific coral reefs to sea-level rise in the 21st century

**DOI:** 10.1098/rsos.150181

**Published:** 2015-07-22

**Authors:** R. van Woesik, Y. Golbuu, G. Roff

**Affiliations:** 1 Department of Biological Sciences, Florida Institute of Technology, Melbourne, FL, USA; 2 Palau International Coral Reef Center, PO Box 7086, Koror, PW 96940, Republic of Palau; 3 School of Biology, University of Queensland, St Lucia, Queensland, Australia

**Keywords:** climate change, sea-level rise, Pacific, coral reefs, corals

## Abstract

Since the Mid-Holocene, some 5000 years ago, coral reefs in the Pacific Ocean have been vertically constrained by sea level. Contemporary sea-level rise is releasing these constraints, providing accommodation space for vertical reef expansion. Here, we show that *Porites* microatolls, from reef-flat environments in Palau (western Pacific Ocean), are ‘keeping up’ with contemporary sea-level rise. Measurements of 570 reef-flat *Porites* microatolls at 10 locations around Palau revealed recent vertical skeletal extension (78±13 mm) over the last 6–8 years, which is consistent with the timing of the recent increase in sea level. We modelled whether microatoll growth rates will potentially ‘keep up’ with predicted sea-level rise in the near future, based upon average growth, and assuming a decline in growth for every 1°C increase in temperature. We then compared these estimated extension rates with rates of sea-level rise under four Representative Concentration Pathways (RCPs). Our model suggests that under low–mid RCP scenarios, reef-coral growth will keep up with sea-level rise, but if greenhouse gas concentrations exceed 670 ppm atmospheric CO_2_ levels and with +2.2°C sea-surface temperature by 2100 (RCP 6.0 W m^−2^), our predictions indicate that *Porites* microatolls will be unable to keep up with projected rates of sea-level rise in the twenty-first century.

## 1. Introduction

**Figure 1.**
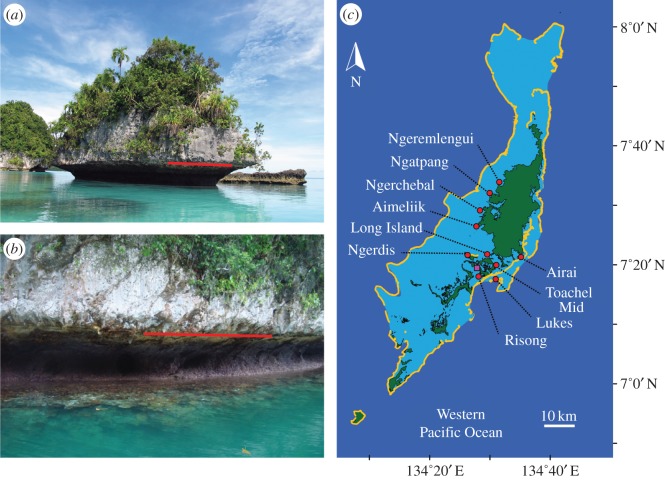
(*a*) Evidence of stable sea level along the ubiquitous limestone islands of Palau, (*b*) notches extend approximately 2–3 m horizontally at lowest spring tide (red line, highest tide mark), (*c*) study sites in Palau, western Pacific Ocean (dark blue, open ocean; pale blue, lagoon; yellow outline, outer reef locations; green, land; tide data from Malakal tide gauge marked with a purple square).

A rapid rise in sea level followed the last ice age, around 18 000 years ago, when sea level was approximately 130 m lower than today [1]. On a global scale, sea-level rise was not homogeneous, but was dependent on local and regional isostatic rebound effects and regional tectonics [1,2]. Depending on their locality, coral reefs either: (i) kept up with sea-level rise, (ii) eventually caught up with sea level, or (iii) drowned [3]. Sea level stabilized in the Pacific Ocean around 5500 years ago [2]. Evidence of this static sea level is apparent along many limestone islands in the Pacific Ocean, including Palau (Micronesia), where notches extend approximately 3 m horizontally at sea level (figure 1*a*,*b*). These notches have been eroded through the millennia by freshwater and reduced alkalinity caused by terrestrial run-off, and suggest that tectonic subsidence has been minimal through the Late-Holocene [4]. Since the Mid-Holocene, reef flats have been constrained by low water spring tides at modern sea level and have existed largely in a dormant state [5,6].

The unprecedented burning of fossil fuels following the industrial revolution in the eighteenth century, and the rapid increases in atmospheric carbon dioxide (CO_2_) and other greenhouse gas levels over the last 50 years have resulted in increasing atmospheric and ocean temperatures, melting ice sheets and rising sea levels [7]. As a consequence, coral reefs throughout the world have suffered thermal-stress events that have caused considerable coral mortality [8], and rising sea levels are resulting in an increase in vertical accommodation space, particularly on reef flats [5,7]. Yet, whether reefs will keep up with modern sea-level rise is largely unknown.

As a small island developing nation in the western Pacific Ocean, rising sea levels represent a significant threat to the economy and ecosystems of Palau [9]. Here, we examine the response of coral reefs to contemporary sea-level rise in Palau (figure 1*c*). In patch reefs and fringing reefs throughout Palau and in the western Pacific Ocean, massive forms of *Porites*species have been the primary reef-framework builders throughout the Late-Holocene, and a high and consistent density of massive *Porites* colonies are most typical of near-shore habitats in the tropical Pacific Ocean [4,10,11]. These coral colonies grow rapidly, and accrete framework at rates of approximately 1 m in 100 years [4]. Despite recent declines in calcification over the past decades [12,13], massive *Porites* are relatively resilient to both decreasing pH [14,15] and increasing temperature [16,17], representing ‘winners’ under future climate-change scenarios. Therefore, as in the past, the ability of reef flats to keep up with rising sea levels will be largely dependent on the persistent high densities and vertical extension of reef-framework species such as massive *Porites*.

**Figure 2.**
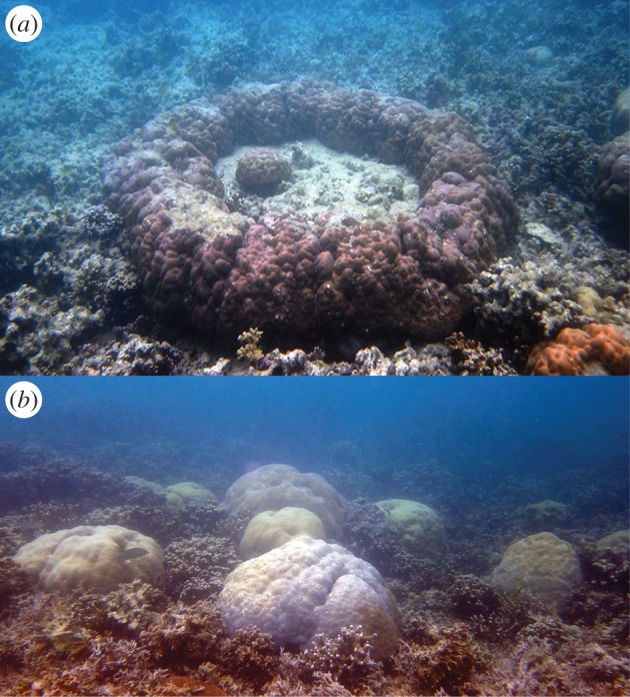
(*a*) In intertidal reef-flat environments, massive *Porites* form characteristic ‘microatoll’ formations, with living tissues around the perimeter, and dead skeleton on the exposed upper surface. Microatoll growth is predominantly lateral, as vertical growth is limited by a lack of accommodation space. (*b*) In sub-tidal reef environments (approx. 1–2 m depth), massive *Porites* are unconstrained by sea level, and form dome shaped colonies through lateral and vertical growth.

In intertidal reef-flat environments, massive *Porites* form characteristic ‘microatoll’ formations (figure 2), expanding laterally, but constrained by aerial exposure at low water spring tides. Consequently, microatolls have been previously used as natural recorders of past and present sea-level changes [18,19]. We explored long-term trends in sea-level rise in Palau over the past century using tide gauge data. To investigate the response of reef flats to the potential increase in recent accommodation space in Palau, we measured recent vertical extension (growth) of *Porites* ‘microatolls’ from 10 lagoonal-patch and fringing reef locations in June 2014 (figure 1*c*). We hypothesized that synchronous vertical extension of *Porites* microatolls across all sites, without any recent tectonic activity in Palau [4], would be evidence of recent adjustment to modern sea-level rise. We then modelled whether microatoll growth rates will potentially ‘keep up’ with predicted sea-level rise in the near future under different climate-change scenarios.

## 2. Material and methods

### 2.1 Sea-level data

**Figure 3.**
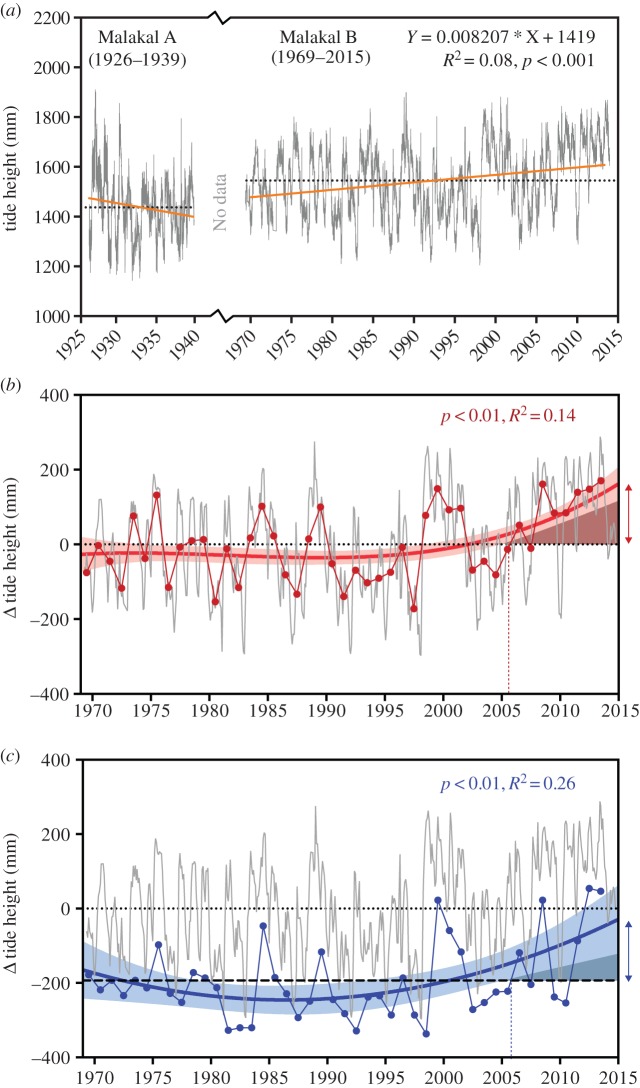
(*a*) Local daily tide data (grey line) from Malakal A (1926–1939) and Malakal B (1969–2015) fitted with linear regression (orange line), showing a 2.87 mm increase per year since 1969 in the Malakal B time series; dotted black line is the mean for each time period. (*b*) Tide data from Malakal B showing monthly averages (grey line), annual averages (red points) and long-term increase above the 1969–2015 average indicated by the significant cubic fit (red curve ± 95% CIs); double-headed red line shows recent difference in tidal height from average, the red-dashed vertical line signifies the approximate timing of the onset of *Porites* vertical extension. (*c*) Tide data from Malakal B showing monthly averages (grey line), annual mean low water spring tide height (blue points) and long-term increase in mean low water spring tide height above the 1969–2015 average (thick dashed black line) indicated by the significant quadratic fit (blue line ± 95% CIs); double-headed blue line shows recent accommodation space, the blue dashed vertical line signifies the approximate timing of the onset of *Porites* vertical extension.

Sea-level data were obtained from a nearby tide gauge, located central to our study sites for two available time periods: (i) 1926–1939 (Malakal A), and (ii) 1969–2015 (Malakal B) [20] (figure 3). A simple linear regression and a polynomial analysis were undertaken of 45-year monthly tidal data using ‘lm’ in the R-base package [21] to determine the lines of best fit and the 95% CIs. The best polynomial fits were determined using Akaike information criterion values. The time-series analyses examined trends in the monthly averages, annual tidal averages and annual mean low water spring tide heights.

### 2.2 Microatoll growth measurements

**Figure 4.**
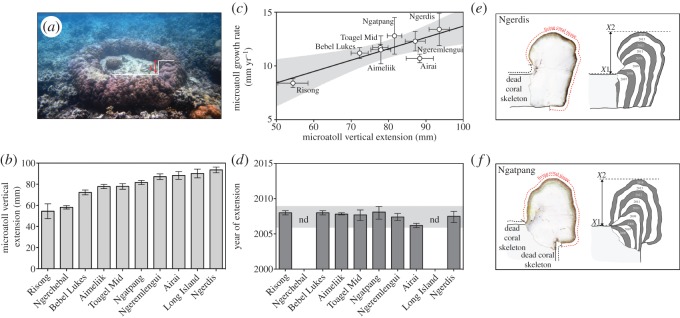
(*a*) High tide across a reef flat in Palau showing field measurement protocol, (*b*) average vertical growth of *Porites* microatolls at each of 12 sites (means ± s.d.), (*c*) relationship between microatoll growth rate and vertical extension of *Porites* microatoll rims (means ± s.d.) and the shaded polygon is the 95% CI, (*d*) hindcasted timing of the onset of microatoll-rim extension (means ± s.d.) and the shaded polygon is the 95% CI, and *Porites* microatoll rim sections from the (*e*) Ngerdis and (*f*) Ngatpang sites, showing a cross section of the skeleton, and a schematic interpretation of the annual growth bands derived from X-rays (*X*1, initial height constrained by sea level; *X*2, height of vertical extension unconstrained by sea level).

To test our hypothesis of recent evidence of spatially consistent vertical extension of *Porites* microatolls, we surveyed microatolls from 10 sites around Palau in June 2014 (figure 1). At each site, 60 randomly chosen *Porites* microatoll colonies were measured for vertical extension (at Long Island, only 30 microatolls were measured). A straight-edge steel bar was placed atop of each living *Porites* colony, suspended by the living edges of the microatoll. The vertical distance from the bottom of the straight edge to the highest point on the microatoll was measured to the nearest mm (figure 4*a*). To explore patterns of growth rates in microatolls, we obtained slabs of microatoll rims from eight of the 10 sites, which were not located in marine protected areas. To explore annual patterns in microatoll growth rates, we sectioned the microatoll rims (figure 4) (*n*=3 sections per site, 7 mm thick sections), and used X-rays to reveal annual growth bands in the skeletons and to quantify annual growth rates [18]. All data analyses were undertaken in R [21].

### 2.3 Model of microatoll growth

To consider whether microatoll growth rates can ‘keep up’ with predicted sea-level rise in the near future, we estimated microatoll extension rates from 2014 to 2100 based upon: (i) average growth of 11.8 mm based upon X-ray measurements of *Porites* microatolls, and (ii) assuming a decline in growth of 41–56% for every 1°C increase in temperature for *Porites* corals ([22]; see the electronic supplementary material). Our model also assumed a subsidence rate of 0.55 mm per year based upon the regional tectonics [23], and that reef accretion is directly related to linear extension of microatolls. This assumption is not unreasonable considering that patch reefs and lagoonal reefs in Palau have been dominated by *Porites* for millennia [4]. We then compared these estimated extension rates with rates of sea-level rise under four Representative Concentration Pathways (RCPs) (2.6, 4.5, 6.0 and 8.5 W m^−2^) [7].

## 3. Results

Average tide levels were broadly comparable between the two datasets, with slightly lower average tides in the 1926–1939 period than in the 1969–2014 period (figure 3*a*). A simple linear analysis of the 45-year daily tidal data since 1969 showed an increase in sea-level of 2.87 mm yr^−1^ (figure 3*a*). This increase is consistent with the regional Pacific average of 2.7±0.6 mm yr^−1^ (1970–2008) and is higher than the global average of 1.8±0.5 mm yr^−1^ (1970–2008) [24,25]. Sea-level rise was nonlinear, however, and both average tidal height and mean low water springs increased after approximately 2006 (figure 3*b*,*c*), resulting in an increase of approximately 160 mm in accommodation space after several millennia of stasis.

All 570 intertidal *Porites* microatolls measured in the field exhibited a flat-surface profile with evidence of recent vertical extension of each colony's rim (figure 4*a*). This growth form is consistent with a long period of sea-level stasis, followed by a recent period of sea-level rise [18]. By contrast, sub-tidal *Porites* assumed a massive growth form because they were not constrained by aerial exposure at low spring tides (figure 2). Vertical extension of the microatoll rims varied among sites, ranging from 54 to 93.5 mm (average 78±13 mm, figure 4*b*), which is consistent with the recent increase in sea level since 2006 (approx. 160 mm). The recent vertical extension of microatolls appeared to be limited by their intrinsic growth rate (figure 4*c*), rather than the availability of accommodation space (figure 3).

Microatoll growth rates varied among sites from 8.4 to 13.4 mm per year (figure 4*c*). These geographical differences were probably a consequence of differential exposure and differences in average flow rates among sites; for example, the highest growth rates were recorded at Ngerdis, which was consistently well flushed, whereas the lowest growth rates were recorded at Risong, which was a calm, sheltered bay that was leeward of a channel (figure 4*b*). Yet, the timing of the onset of vertical extension was remarkably consistent among sites, occurring between 2006 and 2008 (figure 4*d*). The initiation of the microatoll rim extensions coincided with the approximately 160 mm rise in sea level since 2006 (figure 4*e*,*f*).

**Figure 5.**
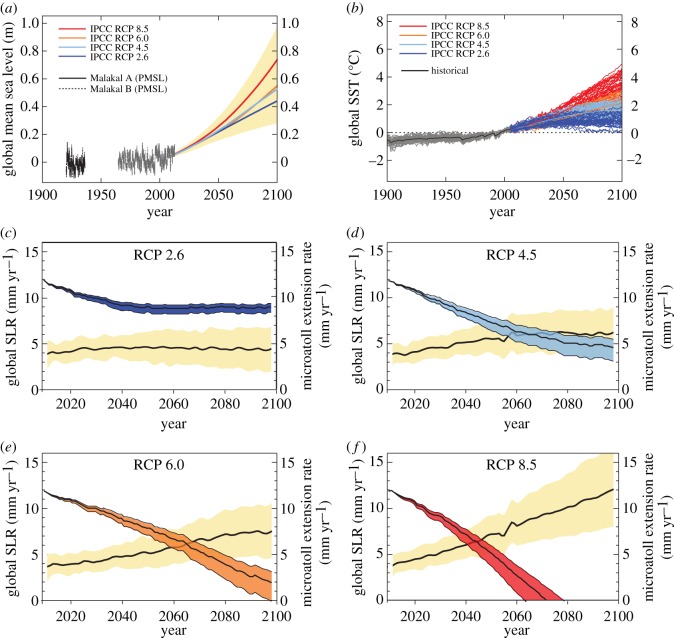
(*a*) Measured tide gauge data from Malakal, Palau and projected global mean sea level (m) (relative to 1984–2005) for four different IPCC (Intergovernmental Panel on Climate Change) RCP (Representative Concentration Pathway) scenarios (2000–2100); (*b*) Global sea-surface temperature (SST °C) past and projected increases for four different IPCC RCP scenarios (2000–2100); and for (*c*–*f*), projected rates of global sea-level rise (SLR) (mm yr^−1^) for four different IPCC RCP scenarios (2000–2100) and modelled estimates of massive *Porites* growth rates assuming an average of 51% reduction in growth rates for every 1°C rise in SST (41–56% upper and lower bounds), where (*c*) depicts RCP 2.6, (*d*) depicts RCP 4.5, (*e*) depicts RCP 6.0 and (*f*) depicts RCP 8.5.

The predictive models suggest that under the most conservative emissions pathway (RCP 2.6), sea-level rise is predicted to be minimal, and microatoll growth rates will keep up with sea-level rise by 2100 (figure 5*c*). Under the RCP 4.5 pathway, emissions will peak around 2040 and then decline [7], and there is enough uncertainty in the models that microatolls may still maintain the capacity to keep up with sea-level rise (figure 5*d*). Under RCP 6.0, emission rates will peak in 2080 and then decline [7], and the rates of sea-level rise are predicted to exceed rates of microatoll extension between the years 2050 and 2070 (figure 5*e*). Yet, there is also some uncertainty in the models under RCP 6.0, and microatolls may still maintain the capacity to keep up with sea-level rise. Under the RCP 8.5 pathway, sea-level rise will exceed 12 mm yr^−1^, increasing temperatures will result in complete impairment of microatoll growth by 2050 (figure 5*f*), and reef flats will not be able to keep up with rising sea levels.

## 4. Discussion

The contemporary growth rates of *Porites* microatolls on the reefs of Palau ranged from 8.4 to 13.4 mm yr^−1^, and averaged 11.8 mm yr^−1^. These growth rates are consistent with growth rates of *Porites* on the inner (10–18 mm yr^−1^) and mid-shelf reefs (6–12 mm yr^−1^) of the Great Barrier Reef in the late 1970s [26]. A previous space-for-time study on the Great Barrier Reef showed that increases in average sea-surface temperatures would increase the average annual extension rates of *Porites* by 3.1 mm for each 1°C rise in temperature [27]. Such space-for-time approaches are useful but do not consider local adaptations. Bearing in mind these caveats, the optimal temperature threshold for coral growth may have been already surpassed on the inner Great Barrier Reef [28]. Recent increases in temperatures appear to have caused the slowing of coral growth rates on the near shore Great Barrier Reef [12,13,22,29] (see also the electronic supplementary material), from an average of 15.2 mm yr^−1^ in 1988 to 12.8 mm yr^−1^ in 2003, a decline of 1.02% yr^−1^, which has been attributed to a corresponding increase in sea-surface temperature [12].

The water temperatures around the islands of Palau range from 27.5 to 31.5°C (Y.G. 2014, unpublished data) and although the large, extant, massive *Porites* bleached during the anomalous thermal-stress events of 1998 [30] and 2010 [31], they survived both events, although the temperatures for optimal massive *Porites* growth rates may have been already surpassed in Palau (see the electronic supplementary material). The near-shore reefs around Palau also experience relatively low pH conditions [15,32], with apparently little consequential effect on coral-community structure and on calcification rates, but bioerosion rates are reported to be high [32]. Despite these low-pH conditions and thermal-stress events, the present results provide evidence of vertical extension of twenty-first century *Porites* microatolls in response to modern sea-level rise. It is still unknown, however, whether *Porites* growth will be able to ‘keep up’ with continued sea-level rise. Keeping up with sea-level rise will be largely dependent on: (i) future rates of sea-level rise, and (ii) future responses of microatoll growth in a warmer ocean.

The rate of sea-level rise is expected to increase substantially into the twenty-first century [7], with estimates of sea-level rise between 36 and 81 cm by 2100 as an indirect result of increasing CO_2_ concentrations in the atmosphere (421–936 ppm by 2100) (figure 5*a*,*b*). Concurrently, increases in sea-surface temperatures (1.0–3.7°C by 2100) are projected to cause substantial declines in the rates of coral growth [12,13,22]. Therefore, to ensure the ‘keep up’ status of coral reefs with rising sea levels, reef management will need to strategize to maximize living coral cover, which is commensurate with net reef accretion capacity [3,33]. The uncertainties in the predictive model in this study stem from several unknown relationships, including the relationship between future microatoll growth at high temperatures [28,29,34], and to what degree reef crests will continue to protect massive *Porites*colonies from typhoon waves in lagoons.

As a small high-island nation, Palau is likely to be impacted by projected sea-level rise along its extensive coastline. Socio-economic analyses suggest that while in most countries the annual cost of coastal protection is less than 0.1% of gross domestic product (GDP), the annual costs of protection are estimated to reach 0.2–0.5% of the Palauan GDP by the 2080s, representing a substantial economic burden [9]. Healthy, vertically accreting reefs will considerably relieve that burden. Other low-lying coral islands in the Pacific, such as the Republic of the Marshall Islands, which barely extend more than 1 m above modern sea level, are even more vulnerable than Palau to sea-level rise.

Our results indicate that a reduction in the rapid increase in greenhouse gas emissions, corresponding to the difference between RCPs 8.5 and 6.0 W m^−2^ (i.e. from 936 to 670 ppm of CO_2_), may make the difference between reefs keeping up with sea level or ultimately drowning under rising seas. Focusing management practices on reducing local stressors while reducing CO_2_ emissions at a global scale will allow these self-sustaining biogenic barriers to protect Pacific island nations and their resources from sea-level rise. If coral reef growth cannot ‘keep up’ with sea-level rise, these natural island storm barriers will disappear, resulting in inundation and reductions in the habitable land for millions of people throughout the Pacific Ocean.

## Supplementary Material

Sea level rise Porites Supplementary document June 15 2015.pdf

## Supplementary Material

Final data set Coral growth and Xrays Palau Sea level study June 15 2015.xlsx

## Data accessibility

The datasets and R code used for analysis are available in the electronic supplementary material.
