# Supplementary material for: Keep up or drown: adjustment of western Pacific coral reefs to sea-level rise in the 21st century
Source: R Soc Open Sci. 2015 Jul 22;2(7):150181. doi: 10.1098/rsos.150181 (PMC4632590; doi:10.1098/rsos.150181)
Supplement: Sea level rise Porites Supplementary document June 15 2015.pdf [file rsos150181supp1.pdf]

Supplementary document

Keep up or drown: adjustment of western Pacific coral reefs to sea-level rise in the 21st century

R. van Woesik[1*], Y. Golbuu[2], G. Roff[3],

Palau is located in the western Pacific warm pool (7.35°N), where the average water temperatures range from 27.5 to 31.5°C (Golbuu unpublished data for 2014). Although significant increases in ocean sea surface temperatures over the 20th Century has allowed for increases in *Porites* growth rates in Western Australia (Cooper *et al* 2012), these locations are in high latitudes (17-28°S), and experience lower average temperatures (21.5-27.6°C, [31]) than Palau. To explore the response of massive *Porites* growth rates under a range of average temperatures, we examined 60 different sites for rates of *Porites* extension from 12 locations (Table S1).

Extension rates of massive *Porites* under a range of temperatures shows a unimodal function, with extension rates increasing with increasing temperatures, peaking at 28.9°C, beyond which the extension rates declined (Figure S1). We note that such a space-for-time approach is useful, because it is not often convenient to wait for decades before results, and the knowledge that stems from the results, are applied to future predictions. However, there are also potential problems with such a space-for-time approach, which does not consider long-term spatial adaptations; and therefore the temporal responses may differ from the spatial responses. However, the unimodal response and decline in growth at higher temperatures (Figure S1) are similar to those previously reported for *Porites* calcification rates (Lough & Cantin 2014). For every degree Celsius increase

in temperature beyond 28.9^{o}C, extension rates declined by approximately 4.48 +1.71 mm yr^{-1} (Table S2). These rates are similar to that of Tanzil et al. (2009) used in our model, who estimate that for every 1°C increase in temperature, rates of extension would reduce growth by 41–56 %.

We also note however that some of the Indonesian corals (Edinger et al 2000), collected at high temperature sites may have been influenced by local pollution. However, the relatively slow growth in Palau (the present study) is not a function of local pollution, nor are the clear regional patterns in Southeast Asia (Tanzil et al 2013) a sign of local pollution. Instead, as Tanzil et al (2013) argued, the reductions in growth and calcification are a function of high water temperatures. Lough and Cantin (2014) also recently concluded that recent reductions in growth and calcification of massive *Porites* on the Great Barrier Reef were a direct function of high water temperatures.

Table S1. Linear extension of massive *Porites* corals and average temperatures from 12 locations in the Indo-Pacific

Site	Temp	Extension	Ref
GBR	23.14	3.398	Lough 2008
GBR	23.561	4.335	Lough 2008
GBR	23.746	3.773	Lough 2008
GBR	24.289	5.357	Lough 2008
GBR	24.455	7.217	Lough 2008
GBR	24.566	6.738	Lough 2008
GBR	24.696	5.948	Lough 2008
GBR	25.038	9.357	Lough 2008
GBR	25.148	9.357	Lough 2008
GBR	25.126	8.83	Lough 2008
GBR	25.158	8.387	Lough 2008
GBR	24.806	9.325	Lough 2008
GBR	25.07	11.138	Lough 2008
GBR	25.401	9.229	Lough 2008
GBR	25.394	8.666	Lough 2008
GBR	26.122	13.374	Lough 2008
GBR	26.135	12.175	Lough 2008
GBR	26.262	13.123	Lough 2008
GBR	25.426	12.115	Lough 2008
GBR	25.393	11.255	Lough 2008
GBR	25.456	10.435	Lough 2008
GBR	26.004	8.307	Lough 2008
GBR	26.471	16.536	Lough 2008
GBR	26.564	15.919	Lough 2008
GBR	26.462	15.611	Lough 2008
GBR	26.742	16.119	Lough 2008
GBR	26.762	14.874	Lough 2008
GBR	26.916	14.475	Lough 2008
GBR	26.923	13.681	Lough 2008
GBR	26.758	12.6	Lough 2008
GBR	26.758	12.183	Lough 2008
GBR	26.913	11.909	Lough 2008
GBR	27.051	11.722	Lough 2008
GBR	26.481	12.475	Lough 2008
GBR	26.556	12.475	Lough 2008
GBR	26.556	13.66	Lough 2008
GBR	26.481	13.433	Lough 2008

GBR	26.256	14.728	Lough 2008
GBR	27.044	14.759	Lough 2008
GBR	27.044	15.439	Lough 2008
GBR	27.044	16.731	Lough 2008
GBR	28.737	20.459	Lough 2008
Arabian Gulf	25.472	11.565	Lough 2008
Arabian Gulf	25.824	10.5	Lough 2008
Arabian Gulf	25.824	10.025	Lough 2008
Arabian Gulf	25.829	12.589	Lough 2008
Lihir	29.535	20.433	Lough 2008
Thailand	28.215	20.82	Tanzil et al 2009
Thailand	28.501	20.87	Tanzil et al 2009
Thailand	28.986	20.54	Tanzil et al 2009
Thailand	29.497	19.45	Tanzil et al 2009
Thailand	29.892	15.55	Tanzil et al 2009
Tj Setan	27.1	16.1	Edinger et al 2000
Hila	26.8	15.4	Edinger et al 2000
Wayame	26.5	13.5	Edinger et al 2000
P.Kecil	30.3	16.3	Edinger et al 2000
G.cemara	28.5	16	Edinger et al 2000
Lagun Marican	31	11.7	Edinger et al 2000
Panjang	28.7	13.5	Edinger et al 2000
Palau	29.8	11.8	Palau: This study

Table S2. Comparison of rates of growth of massive *Porites* with increasing water temperatures

Temperature (^{o}C)	Our model (mm/yr)	Tanzil et al (2009) (mm/yr)
29	20	20
30	17.22	11.80
31	14.44	6.96
32	11.66	4.11
33	8.88	2.42
34	6.1	1.43
35	3.32	0.84
36	0.54	0.50
37	-2.24	0.29

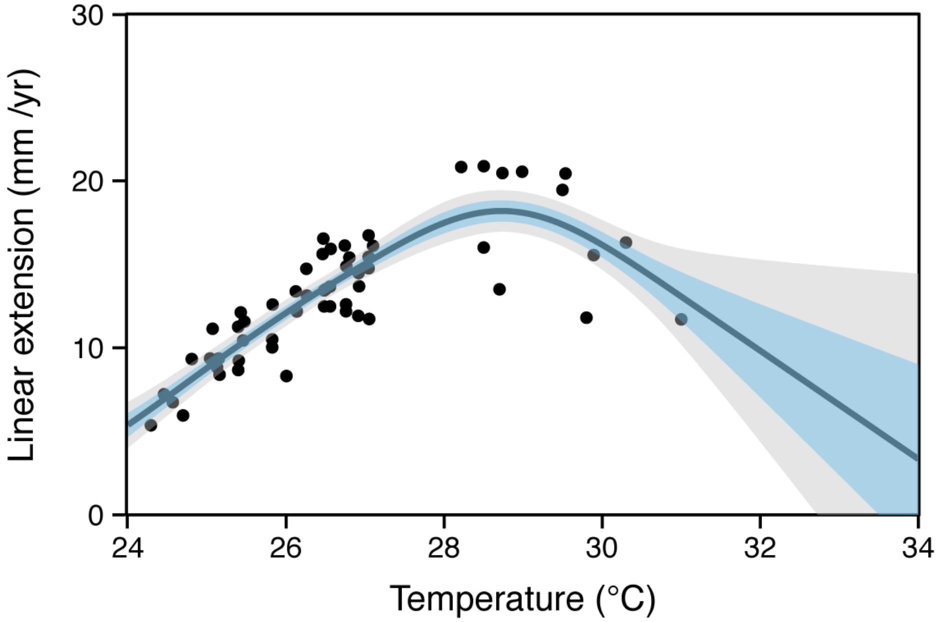

Figure S1. Regression plot of linear extension of massive *Porites* and average water temperature from 60 sites in the Indo-Pacific Ocean, where the grey shade area denotes the 95% confidence intervals, and the blue shaded area denotes the standard errors of the best fit generalized additive model, dark line (using the R package ‘mgcv’).

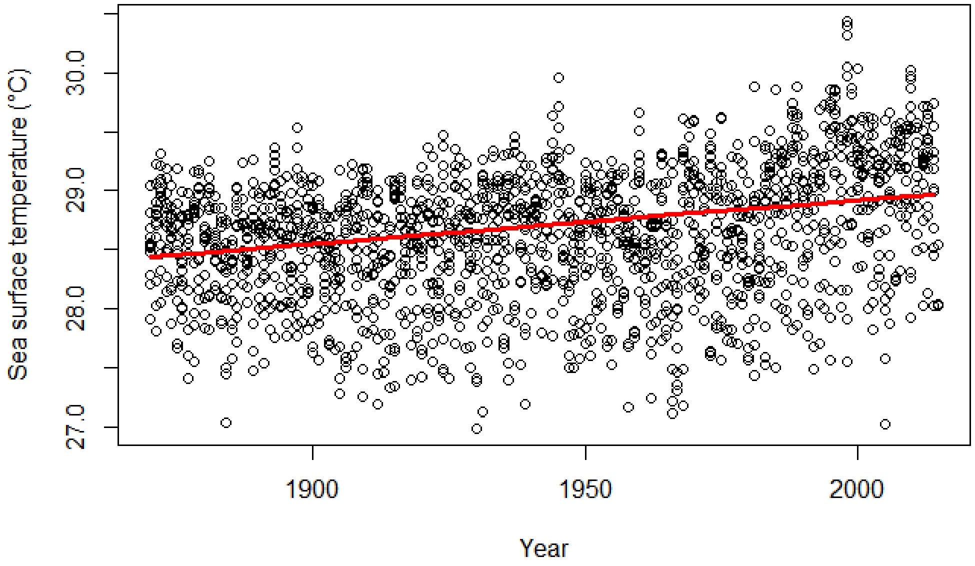

Figure S2. Regression plot of sea surface temperature for Palau dating back to 1870 through to March 2015 using HadISST (http://www.metoffice.gov.uk/hadobs/hadisst/data/). The slope of the regression, or the rate of change in temperature, was estimated at 0.0037 degrees Celsius per year. The code for extracting data and the regression is given in the appendix.

R code for analyzes

```
###############
library(mgcv)
library(ggplot2)
porgro<- read.csv("porgro.csv", header=TRUE)

model2<-gam(Extension~s(Temp), data=porgro)
summary(model2)
testdata = data.frame(Temp=seq(24,36,
length=600),Extension=mean(model2$model$Extension))
fits = predict(model2, newdata=testdata, type='response', se=T)
predicts = data.frame(testdata, fits)
ggplot(aes(x=Temp,y=fit), data=predicts) +
#Confidence intervals
  geom_smooth(aes(ymin = fit - 1.96*se.fit, ymax=fit + 1.96*se.fit),
            colour="black", fill='grey', size=1,stat='identity') +
# Standard errors
  geom_smooth(aes(ymin = fit - se.fit, ymax=fit + se.fit),
             colour="black", fill='#56B4E9', size=1,stat='identity') +
  xlim(24,36) + theme_bw() + ylim(-9,25)+
  xlab(expression(paste("Temperature ("*degree*C*")")))+
                  ylab("Linear extension (mm /yr)")+
  geom_point(aes(x=Temp, y=Extension),data=porgro)

#############################################
#Examine the residuals
fv <- predict(model2,type="terms") ## get term estimates
## compute partial residuals for first smooth...
prsd1 <- residuals(model2,type="working") + fv[,1]
plot(model2, xlim=c(22,34), ylim=c(-18,18),
       xlab = expression(paste("Temperature ("*degree*C*")"), cex = 1.5),
     ylab = list("Residuals of linear extension (proportion)"), shade= TRUE)
ind <- sample(1:length(prsd1),60)
points(porgro$Temp[ind],prsd1[ind],pch=19,col="black")

# Color code
#plot(model2, xlim=c(23.5,34), xlab = expression(paste("Temperature ("*degree*C*")"), cex =
#1.5),
 #    ylab = list("Residuals of linear extension (proportion)"), shade= TRUE)
#ind <- sample(1:length(prsd1),60)
#REF<- as.factor(porgro$Ref)
#points(porgro$Temp[ind],prsd1[ind],pch = c(1, 8,16,25)[as.factor(REF)],
 #      col = c("red", "blue","green", "cyan")[as.factor(REF)]])
```

# Sea surface Temperature

```
library(raster)
#  http://www.metoffice.gov.uk/hadobs/hadisst/data/HadISST_sst.nc.gz
setwd()
#library(R.utils)
#gunzip('HadISST1_sst.nc.gz')
r<-stack('HadISST_sst.nc')
Palau<- SpatialPoints(data.frame(134.582520,7.514980))
#detach("package:R.utils", unload=TRUE)
plot(r,1)
points(Palau)
SST<-extract(r,Palau)
SST<-as.numeric(SST)
#############
#Palau SST 1870 to 2014
rm(list=ls())
setwd("C:/RobsR/SST/Palau")
par(mfrow=c(1,1))
df<- read.csv("C:/RobsR/SST/Palau/Palau Temps.csv", header= TRUE)
plot(df$Year, df$Temp, lty=1, xlab="Year", ylab="SST")
regress <-lm(df$Temp~df$Year)
summary(regress)
coef(regress)
xnew = seq(1870, 2014, 12);
fit = 21.52 + 0.0037*xnew;
plot(df$Year, df$Temp, xlab="Year", ylab="Sea surface temperature (°C)") # original data
lines(xnew, fit, lty=1, col = "red", lwd=3)
```

References

Cooper TF, De 'Ath G, Fabricius KE, Lough JM. 2008 Declining coral calcification in massive *Porites* in two nearshore regions of the northern Great Barrier Reef. *Global Change Biology* **14**, 529-538

Cooper TF, O'Leary RA, Lough JM. 2012 Growth of Western Australian corals in the Anthropocene. *Science* **335**, 593-596

De'ath G, Lough JM, Fabricius KE. 2009 Declining coral calcification on the Great Barrier Reef, *Science* **323**, 116-119

Edinger EN, GV Limmon, J Jompa, W Widjatmoko, JM Heikoop, MJ Risk. 2000 Normal coral growth rates on dying reefs: are coral growth rates good indicators of reef health? *Marine Pollution Bulletin* 40, 404-425

Lough JM, Barnes DJ. 2000 Environmental controls on growth of the massive coral *Porites*. *J. Exp. Mar. Biol. Ecol.* **245**, 225-243

Lough JM. 2008 Coral calcification from skeletal records revisited**.** *Marine Ecology Progress Series* 373, 257–264

Lough JM, Cantin NE. 2014 Perspectives on massive coral growth rates in a changing ocean. *Biological Bulletin* **226**, 187-202

Lough JM. 2008 Coral calcification from skeletal records revisited**.** *Marine Ecology Progress Series* 373, 257–264

Tanzil JTI, Brown BE, Tudhope AW, Dunne RP. 2009 Decline in skeletal growth of the coral *Porites lutea* from the Andaman Sea, South Thailand between 1984 and 2005. *Coral Reefs* **28**, 519-528

Tanzil J.T.I., Brown B.E., Dunne R.P, Lee J.N., Kaandorp J.A., Todd P.A (2013) Regional decline in growth rates of massive Porites corals in Southeast Asia. Global Change Biology 19: 3011-3023
